# Unmet needs in PKU and the disease impact on the day-to-day lives in Brazil: Results from a survey with 228 patients and their caregivers

**DOI:** 10.1016/j.ymgmr.2020.100624

**Published:** 2020-07-22

**Authors:** Ana Maria Martins, Andre Luiz Santos Pessoa, Andrea Amaro Quesada, Erlane Marques Ribeiro

**Affiliations:** a Reference Center in Inborn Errors of Metabolism, Universidade Federal de São Paulo, São Paulo, Brazil; b Albert Sabin Children's Hospital, Fortaleza, Ceará, Brazil; c State University of Ceará (UECE), Fortaleza, Ceará, Brazil; d Edson Queiroz Foundation University of Fortaleza (UNIFOR), Fortaleza, Brazil; e Christus University Center Medical School, Fortaleza, Brazil

**Keywords:** Phenylketonuria, PKU, Phenylalanine, Newborn screening, Brazilian population, Inborn errors of metabolism, PKU, Phenylketonuria, Phe, phenylalanine, PAH, phenylalanine hydroxylase, PEG-PAL, pegylated recombinant phenylalanine ammonia lyase, NBS, Newborn Screening, FDA, Food and Drug Administration, EMA, European Medicines Agency

## Abstract

**Background:**

Accumulation of phenylalanine (Phe) due to deficiency in the enzyme phenylalanine hydroxylase (PAH), responsible for the conversion of Phe into tyrosine leads to Phenylketonuria (PKU), a rare autosomal recessive inborn error of metabolism with a mean prevalence of approximately 1:10,000 to 1:15,000 newborns. Physical, neurocognitive and psychiatric symptoms include neurodevelopmental disorder as intellectual disability and autism spectrum disorder. The most common treatments such as low-Phe diet and supplements may decrease blood Phe concentrations, but neuropsychological, behavioral and social issues still occur in some patients. This study aimed to better understand (i) the Brazilian population's knowledge about newborn screening (NBS), the main diagnostic method for PKU, as well as (ii) the impacts of phenylketonuria in the daily lives of patients and parents.

**Methods:**

Two surveys in Real World Data format gathering of Brazilian residents by online questionnaires with (i) 1000 parents of children up to 5 years old between March and April 2019; (ii) 228 PKU patients and caregivers in March 2019. The survey was conducted in partnership with Abril Publisher and two Brazilian patient associations: Metabolic Mothers and SAFE Brasil, for families with rare diseases and PKU patients, respectively.

**Results:**

The first questionnaire shows that 93% of parents recognize the importance of NBS and 92% report that their children have undergone the test. Still, two out of ten participants did not know what the exam is or what it is for. From the second questionnaire nine out of ten patients had their PKU diagnosis by NBS. Although strict dietary controls for PKU were claimed by 44% of respondents from second questionnaire, 55% assume not following all nutritionist recommendations and 52% did not maintain routinely Phe control levels. In addition, 53% said they had high spending on medical appointments, therapies and purchase of special foods.

**Conclusions:**

Despite the lack of understanding, the awareness of NBS importance is present in the studied population. The early diagnosis of most PKU patients in the study corroborates with neonatal screening central role of PKU early detection. The difficulty in adhering to dietary adjustments and the possibility that current and new therapeutic strategies other than diet could be determinant to achieve the recommended Phe levels.

## 1 Introduction

Phenylketonuria (PKU; McKusick #261600) is a rare autosomal recessive inborn disorder caused by a mutation in the gene encoding phenylalanine hydroxylase (PAH), which is responsible for the transformation of phenylalanine (Phe) into tyrosine [1]. Impairment of PAH activity leads to the accumulation of Phe in the blood [2], that if untreated can cause irreversible damage to the nervous system, resulting in neurodevelopmental disorders such as intellectual disability, autism spectrum disorder, motor deficits, developmental problems, psychiatric symptoms, among others [1,3,4]. The prevalence of PKU varies worldwide [1,[5], [6], [7], [8], [9], [10], [11]], and Brazil has one of the lowest incidence in Latin America, with approximately 1: 15,000 to 1: 25,000 [12,13].

The diagnosis of PKU by the newborn screening (NBS) allows an early intervention which is of utmost importance for the prevention of neurological damage and mental disability. In Brazil, NBS was implemented in 1976 and became mandatory in 1990, allowing costless NBS in the public healthcare system [14]. Later in 2001, the National NBS Program provided a healthcare network for newborns [15,16]. Unfortunately, despite its importance to identify the mutation causing the PKU, the molecular analysis is not available for a complete PKU diagnosis in the public setting, which could impair the adequate management of patients [16,17].

The primary treatment for PKU in Brazil is a lifelong diet prescribed individually according to the Phe tolerance and nutritional demands as recommended by the Brazilian, American, and European guidelines [[18], [19], [20]]. Although the Phe intake restriction can prevent severe intellectual disability, some levels of impairments in the executive functions are still observed in patients under diet control [21]. Therefore, pharmacological treatments and enzyme replacement therapy may be required for some PKU patients, such as sapropterin [18,22,23] and pegvaliase [24,25]. Recently approved by the FDA and EMA, but still not approved in Brazil, pegvaliase reduces PHE levels regardless the PAH activity with an overall safety and efficacy observed in adults [[24], [25], [26], [27]].

In this context, real-world evidences on social awareness regarding NBS, the unmet medical needs, and impact of PKU in patient and caregiver's daily lives are essential to improve policy planning for this rare disease management. Therefore, we conducted two online surveys to assess (i) the perception of NBS importance by Brazilian caregivers; (ii) the impact of PKU on the daily lives of patients and caregivers; and (iii) the main difficulties faced by PKU patients and their caregivers.

## 2 Material and methods

### 2.1 Study design

Online questionnaires were developed in partnership with Abril Publisher and two Brazilian Rare Diseases Patient Associations: Association Friends of Phenylketonurics (*Associação Amiga Dos Fenilcetonúricos* – SAFE Brasil) and Metabolic Mothers (*Mães Metabólicas)*, both with the endorsement of Brazilian Society of Newborn Screening Inborn Errors of Metabolism (*Sociedade Brasileira de Triagem Neonatal Erros Inatos do Metabolismo* – SBTEIM).

For the NBS questionnaire, participants were identified through the Abril Group's database and received an e-mail invitation with the survey information. The Abril Group's Research Institute also disseminated and applied the questionnaire in its website. For the PKU questionnaires, participants were identified from both Brazilian Rare Diseases Patients Associations database. Those associations disseminated the survey through their social media. Supplementary Table 1 and Supplementary Table 2 shows the questions of NBS and PKU surveys, respectively.

### 2.2 Participants

The NBS questionnaire was answered by 1000 caregivers of children up to 5-year-old from all Brazilian regions between March and April 2019. The PKU questionnaire was answered by 228 PKU patients and caregivers in March 2019. Both surveys were conducted independently, targeting different groups of population. However, since these were an online open access surveys, we cannot exclude the possibility that the same individual has answered both questionnaires. As those questionnaires were answered anonymously, no cross-information was possible.

#### 2.2.1 Variables

Both questionnaires consisted of multiple-choice questions addressing social and demographics data, besides some specific questions regarding the NBS and PKU. Since these surveys were aimed only for descriptive analysis, all data were presented as frequency and no comparison were performed.

#### 2.2.2 Ethics

According to the Brazilian regulation (resolution 510/2016) [28], anonymized surveys of personal opinion do not require approval from the ethics committee. Since these were a public anonymized surveys with a standard disclaimer page at the beginnin of each questionnaire, the Ethics Committee's approval was not required for this study.

## 3 Results

### 3.1 Demographic characteristics

Table 1 describes the social and demographics characteristics of the participants of both surveys. The NBS questionnaire was answered mainly by women of the Southeast region, with low income and high educational level. Conversely, the PKU questionnaire was answered mainly by caregivers, men of the Southeast region, with low income and educational level.

**Table 1:** Demographic characteristics of participants in NBS and PKU questionnaires.

	NBS questionnaire (%) n = 1000	PKU questionnaire (%) n = 228
Gender		
Female	70	49^a^
Male	30	51^a^
Region of residence in Brazil		
North and Midwest	20	8
Northeast	20	11
Southeast	40	55
South	20	26
Family income per minimum monthly salaries		
Up to 3 (Up to R$ 2,994)	45	37
From 3 to 8 (From R$ 2,995 to R$ 6,986)	22	22
From 7 to 15 (From R$ 6,987 to R$ 14,970)	7	8
Above 15 (Over R$ 14,971)	9	8
Did not answered	17	25
Educational level		
No school/some primary school	3	48
Primary school graduate	3	17
Some high school	12	12
High school graduate/Some college	53	13
College degree	29	10
Participants		
Patients with PKU	–	14
Caregiver	–	86
Age		
18–24 years	40	–
25–34 years	37	–
≥35 years	23	–
Child's age		
Under a year-old	44	–
1-year-old	12	–
2-year-old	9	–
3-year-old	7	–
4-year-old	4	–
5-year-old	24	–
Birth setting		
Private hospital	37	–
Public hospital	59	–
At home	4	–

a Refers to the gender of patients with PKU.

### 3.2 Main results of NBS questionnaire

Most of the caregivers (93%) recognize the importance of the test, and 92% report that their child underwent the exam. Still, 38% of participants were not aware of the purpose of the exam. In fact, only 5% of respondents were able to describe the diseases screened and 53% was unaware of the conditions assessed in the standard NBS. In addition, 54% considered that hospitals did not clearly explained the purpose of the test, showing a lack of clarity at the hospitals. Nevertheless, 35% of the families did not seek information beyond that provided by the hospital about NBS test. Fortunately, only few individuals confuse the NBS test with the baby's foot stamp, a measure made for the birth certificate. According to this survey, 11% of the NBS were positive for any abnormality; unfortunately, 38% of the caregivers reported that did not receive a clear explanation regarding the diagnosis. Considering those caregivers of children who did not undergo newborn screening, 67% affirmed that they did not refuse the exam, but it was still not performed, and 12% was not properly advised, showing a possible gap in awareness and compliance with the NBS protocol. Tables 2 shows the main results of the NBS questionnaire.

**Table 2:** Main results of the NBS questionnaire.

	Participants (%)
All parents n = 1000	
Did your child do the test?	
Yes, the hospital provided orientation and asked me about it before doing it	65
Yes, the hospital did it automatically without asking me	27
No	2
I don't know/I don't recall	6
Parents of children who underwent NBS test (n = 913)	
Sample collection for NBS	
On the day of birth	30
One day after birth	27
Two to five days after birth	24
One week after birth	8
One month or more after birth	1
Do not remember	9
Other	1
First breastfeeding or formula intake before sample collection for NBS	
I don't know/I don't remember	26
Yes	63
No	11
Suggestion of expanded NBS	
Yes, but I refused it	6
Yes, and I had it done	20
It was not offered	26
I don't know what expanded newborn screening is	48
Hospital explanation about NBS purpose	
It was clearly explained	46
It was more or less clearly explained	25
It was not explained very clearly	11
The purpose was not explained	18
NBS results reviewed by the child's pediatrician	
Yes	54%
No	16%
I do not remember	30%
Parents of children who had positive NBS test results n = 101	
Responsible for reporting the positive NBS to parents	
Childbirth Hospital	34
Newborn Screening Reference Service	23
Pediatrician	27
Social assistant	13
Level of explanation about the diagnosis and next steps	
Explained clearly	62
Explained more or less clearly	22
Explained unclearly	8
He did not explain anything and asked us to go to the referral service for NBS in my city.	8
Parents of children who did not do NBS test n = 24	
Did refuse the test and why	
Yes, because I see it as a suffering for the baby	4
Yes, because I had no guidance and I was afraid	12
Yes, because I see no advantages in taking the exam	17
I did not refuse, but still the test was not done	67

Considering those who underwent the NBS, a quarter of caregivers reported not recall when they received the test result, 62% received the test result within 30 days after the sample collection, 10% received after one month, and 3% never received it. Of these, 81% of the caregivers affirmed that the NBS was performed up to 5 days after the birth, and 63% after the first breastfeeding, as recommended by Brazilian Ministry of Health [17]. Although available exclusively in a private setting, only 26% of the caregivers reported that the expanded NBS was offered and most of them accepted. Even though the NBS provides important information regarding congenital diseases, a significant number of caregivers did not report (16%) or recall (30%) that their child's pediatricians have reviewed the exam results. Additional answers can be found in Supplementary Table 3.

### 3.3 Main results of PKU questionnaire

Assessing only the PKU population, the survey indicated that 90% of the patients were diagnosed at birth through the NBS. Considering those patients who had late diagnosis, 61% informed that the NBS was not performed at the birth center, and 76% reported that the pediatrician did not ask for the NBS results. This probably have a greater impact in older patients, since the mandatory and free NBS program was implemented in Brazil only in the 1990´s (24% of the PKU patients included in the survey were more than 21 years old). Table 3 presents the main results for PKU questionnaire.

**Table 3:** Main results of patients, parents and caregivers answers in PKU questionnaire.

	Patients, parents and caregivers (%)
Age of PKU diagnosis. n = 228	
Newborn	90
Between 1 and 5 years old	8
Over 10 years old	2
Reason for the late diagnosis. n = 23 (only for those diagnosed late)	
The results of newborn screening were normal/ negative for phenylketonuria	4
Newborn screening was not done at the birth center	61
Newborn screening results were not received	13
Doctors lack of knowledge	9
Other reasons	13
Current age of PKU patient. n = 21 (caregivers of patients with late diagnosis only)	
Under 2 years old	15
From 3 to 5 years old	20
From 6 to 10 years old	17
From 11 to 15 years old	16
From 16 to 20 years old	8
From 21 to 30 years old	19
Above 31 years of age	5
Pediatrician requested the NBS result. n = 21 (caregivers of patients with late diagnosis only)	
No	76
Yes	24
Routine control of PKU levels. n = 228	
Food for all meals is weighed daily, I calculate the amount of phenyl according to the daily allowance provided by the nutritionist along with the amount of formula	48
Food is weighed whenever possible, but I'm unable to calculate the amount of phenyl for all meals and do not always administer the formula	10
I am neither able to weigh food nor do the calculation of the amount of phenyl, but I am experienced at diet management and I administer the formula when I feel that my family member needs to take it	30
Not much control is done, since I see that my child/family member feels well and I can make the diet flexible	12
Perspective of PKU control. n = 228	
Totally controlled 5	41
4	34
3	21
2	3
1 Not controlled at all	1
Greatest difficulty to continue treatment n = 213 (only those who reported difficulties)	
The public health system does not provide low-protein foods	13
Frequently changing amino acid formulas provided by the public health system	21
Following and controlling diet on a daily basis, due to the level of restrictions	18
There is no medication treating phenylketonuria	9
I am unable to easily find the food suggested by the nutritionist	7
Having a different diet from the rest of the family	4
The medication available to treat PKU is not available from the public health system	4
Social exclusion/ lack of understanding by others	2
Daily weighing of all food	1
Doing a blood test at every doctor's visit	1
Difficulty getting to the doctor's office/ lack of appropriate transportation	1
Little understanding and support from family and friends	1
Difficulty understanding the orientation provided by the physician or nutritionist	0
Adherence to physician's instructions n = 228	
I follow all guidelines	42
I follow most guidelines approximately	33
My doctor does not provide many orientations; I receive most of them from my nutritionist	18
I follow half of the guidelines approximately	6
I follow few guidelines	1
Adherence to nutritionist's guidelines n = 228	
I follow all guidelines	44
I follow most guidelines approximately	43
I follow half of the guidelines approximately	9
I follow few guidelines	2
I don't follow anything	1
I don't regularly see a nutritionist	1
Financial impact of PKU. n = 228	
Yes, because I have incurred significant costs from doctor visits, therapy and the purchase of special food	53
Yes, I had to stop working to take care of my child	41
Yes, because people in my family have stopped working to help me or to help our family member with the disease	8
Yes, because I had to hire someone to help me	6
No, because I am financially comfortable and the disease has not impacted my income	16
Prejudice due to PKU. n = 228	
Yes	62
No	38
Brazilian Government preparation to assist PKU patients. n = 228	
Very prepared	1
Somewhat prepared	37
Not at all prepared	62

Overall, the greatest obstacle for the PKU treatment was the lack of access to low-protein foods in the public setting, which affected 31% of the respondents. Additionally, for 21% of the participants, the frequent change in formula components provided by the public healthcare system also affect treatment adherence. Other important complaint was the difficulties in following the daily diet due to the level of restriction. In fact, only 48% of the PKU patients strictly weigh their food and adhere to the diet provided by the nutritionist. Moreover, the physician and nutritionist's orientations are fully followed only by 42% and 44% of the PKU patients, respectively. Consequently, at least a quarter of patients with PKU do not have a controlled condition. Also, 21% reported intellectual impairment. PKU also represents an important socioeconomic burden: 53% of respondents reported a significant financial impact related to the PKU management, 49% had to stop working to care for the PKU patient, and 6% need to hire a caregiver to assist the PKU patient.

Cognitive and emotional symptoms were the most frequent complaint reported by PKU patients: irritability (78%), anxiety (67%), and lack of concentration (58%). Nevertheless, the absence of neuropsychological care was observed in nearly 70% of the patients, since they have never undergone a cognitive and executive functions tests. Additionally, 39% of the patients did not receive the support of a day-to-day psychologist. When asked about the barriers that the disease imposes to the daily routine, respondents highlighted three points: the limitation on social activities (75%), the impact on professional life (57%), and the effect on self-esteem (53%). Overall, 99% of the participants considered that Brazil is not prepared to assist patients with PKU. More results can be found in Table 4 and 5 of Supplementary material.

## 4 Discussion

Overall, there was a high adherence to the NBS at Brazilian birth centers. Although the exam was considered relevant, the information provided by the health practitioners is far from ideal. Additionally, there was no standardization on the NBS procedure, since an important proportion of the test were not performed in accordance to the Brazilian guideline [15,16]. Even though most diagnoses of PKU were made at birth through the NBS, which allows a prompt intervention, almost a quarter of the PKU patients had an uncontrolled disease. Lack of access to an adequate diet and to drug treatment, along with difficulties to follow diet recommendations, and economic burden may worsen the patient's symptoms. Nevertheless, the absence of an appropriate neuropsychological follow-up is still the reality of the vast majority of the Brazilian PKU patients.

It is important to emphasize that the Brazilian population has almost achieved a universal access to healthcare services, with a NBS program that complies to international guidelines [[29], [30], [31]]. Even so, the estimated coverage of NBS program in Brazil is about 80%, with little improvement over the years [32]. Howson et al. described three main reasons why Latin America has not full coverage: the implementation of a national program in a large country such as Brazil would be very challenging in terms of organization, funding, and logistics; the lack of a centralized national system of screening services; and low *per capita* expenditures on health [32]. The discrepancy of NBS Program coverage throughout the national territory, contributed to the unequal geographical distribution of the participants, since the reduced availability or misinformation of the NBS may lead to individuals with undiagnosed PKU. From the caregiver's perspective, poor communication is an important obstacle for NBS adherence. Indeed, Kohlschütter and van den Bussche reported that the main cause of late diagnosis was the inefficient communication between parents, practitioners, and healthcare centers [33]. Proper orientation, even before child's birth, can change this scenario [34]. A frequent lack of coordinated systems involving healthcare professionals, laboratory staff, and caregivers makes proper communication a challenging task and compromises the best care for the child and their families during the testing, results assessment, and any follow-up needed [35].

In PKU questionnaire, we observed essential gaps in adherence to treatments, since nearly half of patients are not adherent to all physician and nutritionist recommendations, which are based on Brazilian guidelines [20]. Also, significant intellectual impairment can be observed in this study, which enhancing the relevance of improving therapy, especially for patients under ten years old, to optimize this outcome [36]. Although dietary therapy involving Phe restriction and supplementation with reduced or Phe-free amino acid mixtures (“formulas”) is effective preventing severe intellectual disability, complex executive functions impairments are still observed in properly treated patients [21]. The lack of access to low-protein foods and changing “formulas” presented by participants show the inadequacy of public policies and the need to reinforce care for patients with PKU.

Not only difficulty in adhering to nutritional adjustments but also the limitations of diet treatment may indicates that therapeutic strategies beyond diet could make a difference to achieve well-established Phe levels of the patients [21]. Only 25–50% PKU patients responds to sapropterin, a synthetic formulation treatment, since it requires a residual PAH enzyme activity to be effective [18,22,23,37,38]. Additionally, enzyme replacement therapy, which has been established for several of the lysosomal storage disorders, is recently available in United States and Europe for PKU as pegvaliase, a pegylated recombinant phenylalanine ammonia-lyase (PEG-PAL) [24,25,39]. Pegvaliase reduces blood Phe concentrations independent of PAH activity compared with previously available therapeutic alternatives [18]. Although treatment with pegvaliase showed overall safety and efficacy in adults during phase III clinical studies, further research should attend younger patients, those with milder forms of PKU, in combination with sapropterin, and with limited dietary therapy [26,27,40].

Results of cognitive and emotional symptoms corroborates with disease characteristics since treated individuals have a higher incidence of neuropsychological problems, mainly Attention Deficit Disorder with Hyperactivity [5,21,41,42]. In a study with early treated adults with PKU, despite the high variability across participants, about 24% showed cognitive impairment [21]. Although results of previous studies reinforce the need for neuropsychological assistance, it contrasts with the lack of neuropsychological evaluation and support reported by the patients in this research [21,42].

PKU has significant socioeconomic repercussions, which include having parents quit their jobs or hiring a person to help them in daily activities. A study by Wang et al. showed that severe financial burden on patients' families might have, as a consequence, negative impacts on the PKU treatment or variation of blood Phe concentration. Less capital available can influence on the disbursement of all relatives, including food, education, and type of treatment [43]. Therefore, financial support from 10.13039/501100002425Brazilian government may reduce this burden, especially for families with low income. Nonetheless, almost all participants considered the country not prepared to assist PKU patients.

When asked about the barriers that the disease imposes to the routine, respondents tend to highlight three points: the limitation on social activities, the impact on working life, and the effect on self-esteem. Social results corroborate with those found in the literature, where lack of general awareness about the disease also reflected in some misunderstanding by society, including prejudice [1,44,45].

This study has some limitations: data has self-reported and cross-sectional nature, and statements about causality cannot be made from the study results. Thus, there is no way to independently verify variables reported (for example, diagnosis, medications taken) *via* other data sources. There is the potential for respondents to misreport some information due to response bias. Since some questions are retrospective, a memory bias can occur. Despite the relevant number of participants for a rare autosomal recessive disease, selection bias may occur. Sampling bias can also contribute to geographical distribution disparity of the participants, since online survey requires that the participant have access to the internet.

## 5 Conclusion

Although the NBS is widely offered, there is still a need to increase NBS awareness of the entire population and to provide an equal and high-quality service across Brazil. For patients with PKU diagnosis, improvements in neuropsychological evaluation and specialist assistance are required to minimize their cognitive and emotional symptoms. The uncontrolled disease caused by low adherence to the restrictive diet could be overcome by the provision of alternative treatments, such as sapropterin and PEG-PAL. Also, providing financial support and treatment reimbursement would reduce the economic burden of PKU disease, especially for low incoming families. In summary, the results of this research highlighted some unmet needs of PKU patients and should serve as reference for public policies to be revised in order to improve the NBS program adherence and the assistance of PKU patients and caregivers.

## Declarations

The study protocol and amendments were approved by institutional review boards or independent ethics committees at participating institutions. The study was done in accordance with The Code of Ethics of the World Medical Association (Declaration of Helsinki), and with applicable regulatory requirements. An authorizing data release publication disclaimer was applied in the online page survey. No EC applicable.

## Consent for publication

An authorizing data release publication disclaimer was applied in the online page survey. No EC applicable.

## Disclose the following

Receiving reimbursements, fees, funding, or salary from an organization that may in any way gain or lose financially from the publication of the manuscript, either now or in the future.

Holding stocks or shares in an organization that may in any way gain or lose financially from the publication of the manuscript, either now or in the future.

Holding, or currently applying for, patents relating to the content of the manuscript.

Receiving reimbursements, fees, funding, or salary from an organization that holds or has applied for patents relating to the content of the manuscript.

## Funding

The medical writer support for this study was supported by BioMarin Brasil Farmaceutica LTDA. The funder played no role in the PKU survey application and its results.

## Authors' contributions

The authors contributed with the survey results analysis for the medical writer activities. Also, imputed their scientific contributions for the results interpretation, as experts in PKU.

## Appendix A Supplementary data

Supplementary material Image 1

## References

[1] van Wegberg A.M.J., MacDonald A., Ahring K., Bélanger-Quintana A., Blau N., Bosch A.M., Burlina A., Campistol J., Feillet F., Giżewska M., Huijbregts S.C., Kearney S., Leuzzi V., Maillot F., Muntau A.C., van Rijn M., Trefz F., Walter J.H., van Spronsen F.J. (2017). The complete European guidelines on phenylketonuria: diagnosis and treatment. Orphanet. J. Rare Dis..

[2] Fölling A. (1934). Über Ausscheidung von Phenylbrenztraubensäure in den Harn als Stoffwechselanomalie in Verbindung mit Imbezillität. Hoppe. Seylers. Z. Physiol. Chem..

[3] Jahja R., Huijbregts S.C.J., de Sonneville L.M.J., van der Meere J.J., Legemaat A.M., Bosch A.M., Hollak C.E.M., Rubio-Gozalbo M.E., Brouwers M.C.G.J., Hofstede F.C., de Vries M.C., Janssen M.C.H., van der Ploeg A.T., Langendonk J.G., van Spronsen F.J. (2017). Cognitive profile and mental health in adult phenylketonuria: a PKU-COBESO study. Neuropsychology.

[4] Feldmann R., Osterloh J., Onon S., Fromm J., Rutsch F., Weglage J. (2019). Neurocognitive functioning in adults with phenylketonuria: report of a 10-year follow-up. Mol. Genet. Metab..

[5] Mitchell J.J., Trakadis Y.J., Scriver C.R. (2011). Phenylalanine hydroxylase deficiency. Genet. Med..

[6] Waisbren S.E., Noel K., Fahrbach K., Cella C., Frame D., Dorenbaum A., Levy H. (2007). Phenylalanine blood levels and clinical outcomes in phenylketonuria: a systematic literature review and meta-analysis. Mol. Genet. Metab..

[7] Borrajo G.J.C. (2016). Newborn screening for phenylketonuria. J. Inborn Errors Metab. Screen..

[8] Borrajo G.J. (2012). Panorama epidemiológico de la fenilcetonuria (PKU) en Latinoamérica. Acta Pediátrica México.

[9] Borrajo G.J.C. (2007). Newborn screening in Latin America at the beginning of the 21st century. J. Inherit. Metab. Dis..

[10] Albrecht J., Garbade S.F., Burgard P. (2009). Neuropsychological speed tests and blood phenylalanine levels in patients with phenylketonuria: a meta-analysis. Neurosci. Biobehav. Rev..

[11] Cepal N.U. (2016). División de Estadísticas, Statistical Yearbook for Latin America and the Caribbean 2015.

[12] Monteiro L.T.B., Cândido L.M.B. (2006). Fenilcetonúria no Brasil: evolução e casos. Rev. Nutr..

[13] Carvalho T. (2003). Resultados do levantamento epidemiológico da sociedade brasileira de triagem neonatal (SBTN), Rev. Médica Minas Gerais.

[14] Sociedade Brasileira de Endocrinologia e Metabologia (SBEM), Histórico da SBEM (2020). http://www.sbteim.org.br/historico-historico.htm.

[15] Marini de Carvalho T., Pimentel dos Santos H., dos Santos I.C.G.P., Vargas P.R., Pedrosa J. (2007). Newborn screening: a national public health programme in Brazil. J. Inherit. Metab. Dis..

[16] BRASIL. Ministério da Saúde (2001). PORTARIA N^o^ 822, DE 06 DE JUNHO DE 2001, Brazil. http://bvsms.saude.gov.br/bvs/saudelegis/gm/2001/prt0822_06_06_2001.html.

[17] BRASIL. Ministério da Saúde (2016). Triagem Neonatal Biológica: Manual Técnico.

[18] Vockley J., Andersson H.C., Antshel K.M., Braverman N.E., Burton B.K., Frazier D.M., Mitchell J., Smith W.E., Thompson B.H., Berry S.A. (2014). American College of Medical Genetics and Genomics Therapeutics Committee, phenylalanine hydroxylase deficiency: diagnosis and management guideline. Genet. Med..

[19] van Spronsen F.J., van Wegberg A.M., Ahring K., Bélanger-Quintana A., Blau N., Bosch A.M., Burlina A., Campistol J., Feillet F., Giżewska M., Huijbregts S.C., Kearney S., Leuzzi V., Maillot F., Muntau A.C., Trefz F.K., van Rijn M., Walter J.H., MacDonald A. (2017). Key European guidelines for the diagnosis and management of patients with phenylketonuria. Lancet Diabetes Endocrinol..

[20] BRASIL. Ministério da Saúde (2019). Estratégicos, Secretaria de Ciência Tecnologia e Insumos Estrategicos, Protocolo Clínico e Diretrizes Terapêuticas – Fenilcetonúria, Comissão Nac. Inc. Tecnol. No Sist. Único Saúde. http://conitec.gov.br/images/Consultas/Relatorios/2019/Relatrio_PCDT_Fenilcetonria_CP16_2019.pdf.

[21] Palermo L., Geberhiwot T., MacDonald A., Limback E., Hall S.K., Romani C. (2017). Cognitive outcomes in early-treated adults with phenylketonuria (PKU): a comprehensive picture across domains. Neuropsychology.

[22] Feillet F., Bonnemains C. (2013). Phenylketonuria: new treatments. Arch. Pediatr..

[23] Burnett J.R. (2007). Sapropterin dihydrochloride (Kuvan/Phenoptin), an orally active synthetic form of BH4 for the treatment of phenylketonuria. IDrugs.

[24] Levy H.L., Sarkissian C.N., Scriver C.R. (2018). Phenylalanine ammonia lyase (PAL): from discovery to enzyme substitution therapy for phenylketonuria. Mol. Genet. Metab..

[25] Mahan K.C., Gandhi M.A., Anand S. (2019). Pegvaliase: a novel treatment option for adults with phenylketonuria. Curr. Med. Res. Opin..

[26] Thomas J., Levy H., Amato S., Vockley J., Zori R., Dimmock D., Harding C.O., Bilder D.A., Weng H.H., Olbertz J., Merilainen M., Jiang J., Larimore K., Gupta S., Gu Z., Northrup H. (2018). Pegvaliase for the treatment of phenylketonuria: results of a long-term phase 3 clinical trial program (PRISM). Mol. Genet. Metab..

[27] Harding C.O., Amato R.S., Stuy M., Longo N., Burton B.K., Posner J., Weng H.H., Merilainen M., Gu Z., Jiang J., Vockley J. (2018). Pegvaliase for the treatment of phenylketonuria: a pivotal, double-blind randomized discontinuation phase 3 clinical trial. Mol. Genet. Metab..

[28] Ministério da saúde (2016). Conselho Nacional de Saúde, Resolução n^o^ 510, de 07 de Abril de 2016.

[29] Castro M.C., Massuda A., Almeida G., Menezes-Filho N.A., Andrade M.V., de Souza Noronha K.V.M., Rocha R., Macinko J., Hone T., Tasca R., Giovanella L., Malik A.M., Werneck H., Fachini L.A., Atun R. (2019). Brazil’s unified health system: the first 30 years and prospects for the future. Lancet (Lond. Engl.).

[30] Leão L.L., de Aguiar M.J.B. (2008). Triagem neonatal: o que os pediatras deveriam saber. J. Pediatr..

[31] Leão L.L., de Aguiar M.J.B. (2008). Newborn screening: what pediatricians should know. J. Pediatr..

[32] Howson C.P., Cedergren B., Giugliani R., Huhtinen P., Padilla C.D., Palubiak C.S., Santos M.D., Schwartz I.V.D., Therrell B.L., Umemoto A., Wang J., Zeng X., Zhao X., Zhong N., McCabe E.R.B. (2018). Universal newborn screening: a roadmap for action. Mol. Genet. Metab..

[33] Kohlschütter A., van den Bussche H. (2019). Early diagnosis of a rare disease in children through better communication between parents, physicians and academic centers. Z. Evid. Fortbild. Qual. Gesundhwes.

[34] Ong L.M., de Haes J.C., Hoos A.M., Lammes F.B. (1995). Doctor-patient communication: a review of the literature. Soc. Sci. Med..

[35] Evans A., Bonhomme N., Goodman A., Terry S.F. (2018). Newborn screening and health communications. Genet. Test. Mol. Biomarkers.

[36] Michals K., Azen C., Acosta P., Koch R., Matalon R. (1988). Blood phenylalanine levels and intelligence of 10-year-old children with PKU in the National Collaborative Study. J. Am. Diet. Assoc..

[37] European Medicine Agency (2015). Kuvan sapropterin dihydrochloride. Eur. Public Assess. Rep..

[38] B. L.E., M. S., F. C (2009). Responsiveness of Kuvan in PKU patients during the expanded access program and following FDA approval. Mol. Genet. Metab..

[39] Wang R.Y., Bodamer O.A., Watson M.S., Wilcox W.R. (2011). ACMG work group on diagnostic confirmation of Lysosomal storage diseases, Lysosomal storage diseases: diagnostic confirmation and management of presymptomatic individuals. Genet. Med..

[40] Hydery T., Coppenrath V.A. (2019). A comprehensive review of Pegvaliase, an enzyme substitution therapy for the treatment of phenylketonuria. Drug Target Insights.

[41] Blau N., van Spronsen F.J., Levy H.L. (2010). Phenylketonuria. Lancet..

[42] Beckhauser M.T., Beghini Mendes Vieira M., Moehlecke Iser B., Rozone DE Luca G., Rodrigues Masruha M., Lin J., Luiz Streck E. (2020). Attention deficit disorder with hyperactivity symptoms in early-treated phenylketonuria patients. Iran. J. Child Neurol..

[43] Wang L., Zou H., Ye F., Wang K., Li X., Chen Z., Chen J., Han B., Yu W., He C., Shen M. (2017). Household financial burden of phenylketonuria and its impact on treatment in China: a cross-sectional study. J. Inherit. Metab. Dis..

[44] Huijbregts S.C.J., Bosch A.M., Simons Q.A., Jahja R., Brouwers M.C.G.J., De Sonneville L.M.J., De Vries M.C., Hofstede F.C., Hollak C.E.M., Janssen M.C.H., Langendonk J.G., Rubio-Gozalbo M.E., Van der Meere J.J., Van der Ploeg A.T., Van Spronsen F.J. (2018). The impact of metabolic control and tetrahydrobiopterin treatment on health related quality of life of patients with early-treated phenylketonuria: a PKU-COBESO study. Mol. Genet. Metab..

[45] Hendrikx M.M.T., van der Schot L.W.A., Slijper F.M.E., Huisman J., Kalverboer A.F. (1994). Phenylketonuria and some aspects of emotional development. Eur. J. Pediatr..

