# Supplementary material for: Unmet needs in PKU and the disease impact on the day-to-day lives in Brazil: Results from a survey with 228 patients and their caregivers
Source: Mol Genet Metab Rep. 2020 Jul 22;24:100624. doi: 10.1016/j.ymgmr.2020.100624 (PMC7387838; doi:10.1016/j.ymgmr.2020.100624)
Supplement: Supplementary file 1 — Supplementary material [file mmc1.docx]

**SUPPLEMENTARY MATERIAL**

**NBS Questionnaire**

- **Original Portuguese version**

**Informações gerais**

1. Gênero ☐ masculino ☐ feminino

2. Idade ☐ 18-24 ☐ 25-34 ☐ 35 ou mais

3. Classe social ☐ A e B ☐ C ☐ D e E

4. Qual é a idade do seu filho? ☐ Menos de 1 ano ☐ 1 ano ☐ 2 anos ☐ 3 anos ☐ 4 anos ☐ 5 anos

5. Onde seu filho nasceu? ☐ Hospital particular ☐ Hospital público ☐ Em casa

6. Em qual região do país você mora? ☐ Norte ☐ Nordeste ☐ Centro-Oeste ☐ Sudeste ☐ Sul

7. Renda familiar ☐ Até R$ 2 994 Três salários mínimos ☐ De R$ 2 995 a R$ 6 986 De três a sete salários mínimos ☐ De R$ 6 987 a R$ 14 970 De sete a quinze salários mínimos ☐ Acima de R$ 14 971 Mais de quinze salários mínimos ☐ Prefiro não responder

8. Escolaridade ☐ Analfabeto/Fundamental incompleto (parou entre o 1º e o 4º ano) ☐ Fundamental I completo (fez até o 5º ano) Fundamental II incompleto (parou entre o 6º e o 8º ano) ☐ Fundamental completo (fez até o 9º ano) Médio incompleto (parou entre o 1º e o 2º colegial) ☐ Médio completo (fez até o 3º colegial) Superior incompleto (começou a faculdade/graduação) ☐ Superior completo (terminou a faculdade/graduação)

**Questionário**

1. Quanto considera importante a realização do teste do pezinho? ☐ Muito importante 5 ☐ 4 ☐ 3 ☐ 2 ☐ 1/0 Nada importante

2. Qual é a frase que melhor descreve o teste do pezinho? ☐ Retirada de algumas gotas de sangue do pé do bebê para diagnóstico precoce de doenças genéticas e/ou metabólicas ☐ Carimbo dos pezinhos entregue na maternidade/hospital pouco depois do Nascimento ☐ Coleta de material genético do pé do bebê para detectar síndrome de own

3. O hospital explicou a finalidade do exame? ☐ Explicou de forma clara ☐ Explicou de forma mais ou menos clara ☐ Não explicou a finalidade ☐ Explicou de forma pouco clara

4. Seu filho fez o teste do pezinho? ☐ Sim, e o hospital me orientou e consultou antes de fazer ☐ Sim, e o hospital fez automaticamente sem me consultar ☐ Não fez ☐ Não sei/ não lembro

5. Chegou a procurar informações sobre o teste do pezinho além do que o hospital ofereceu? Onde? ☐ Profissional de saúde ☐ Hospital ☐ Imprensa em geral ☐ Sites especializados ☐ Redes sociais ☐ Outros pais e mães

6. Quando o teste do pezinho foi feito? ☐ No dia do Nascimento ☐ Um dia após o Nascimento ☐ De dois a cinco dias após o Nascimento ☐ Uma semana após o Nascimento ☐ Um mês ou mais após o Nascimento ☐ Não lembro

7. Seu filho já tinha realizado a primeira mamada com leite materno ou com fórmula quando fez o teste do pezinho? ☐ Não sei/ não lembro ☐ Sim ☐ Não

8. Foi oferecido o teste do pezinho expandido? ☐ Sim, mas eu não aceitei fazer ☐ Sim, e eu aceitei fazer ☐ Não foi oferecido ☐ Não sei o que é o teste do pezinho expandido

9. Após quanto tempo depois da coleta você recebeu o resultado do teste do pezinho? ☐ Até quinze dias após a coleta ☐ De 16 a 30 dias após a coleta ☐ De 31 a 60 dias após a coleta ☐ Mais de dois meses após a coleta ☐ Não recebi os resultados ☐ Não lembro

10. Conhece ou já ouviu falar sobre as principais doenças detectadas pelo teste do pezinho? ☐ Deficiência de biotinidase ☐ Doença falciforme ☐ Fenilcetonúria (PKU) ☐ Fibrose cística ☐ Hiperplasia adrenal congênita ☐ Hipotireoidismo congênito

11. Você sabia que o teste do pezinho está disponível na rede pública e é oferecido de forma gratuita a todos os nascidos vivos no Brasil? ☐ Sim ☐ Não

12. Você recusou a realização do teste do pezinho? ☐ Sim, porque vejo como um sofrimento para o bebê ☐ Sim, porque não tive orientação e tive receio ☐ Sim, porque não vejo vantagens na realização do exame ☐ Não recusei, mas ainda assim o teste não foi feito

13. O pediatra do seu filho pediu para ver o teste do pezinho em algum momento? ☐ Sim ☐ Não ☐ Não lembro

14. O teste do pezinho do seu filho apresentou alguma alteração? ☐ Sim ☐ Não

15. Quem entrou em contato para comunicar que o teste havia dado resultado alterado? ☐ Serviço de referência em triagem neonatal ☐ Hospital em que meu filho nasceu ☐ Pediatra ☐ Assistente social ☐ Outra pessoa

16. Quanto considera que a pessoa que entrou em contato com você explicou claramente o diagnóstico e quais eram os próximos passos? ☐ Explicou de forma clara ☐ Explicou de forma mais ou menos clara ☐ Explicou de forma pouco clara ☐ Não explicou nada e pediu que nos dirigíssemos ao serviço referência em triagem neonatal de minha cidade

- **English version**

**General information**

1 Genre ☐ male ☐ female

2 Age ☐ 18-24 ☐ 25-34 ☐ 35 or over

3 Stratum ☐ A and B ☐ C ☐ D and E

4 How old is your child? ☐ Under a year old ☐ 1 year ☐ 2 years ☐ 3 years ☐ 4 years ☐ 5 years

5 Where was your child born? ☐ Private hospital ☐ Public hospital ☐ At home

6 Which Brazilian region do you live in? ☐ North ☐ Northeast ☐ Midwest ☐ Southeast ☐ South

7 Family income ☐ Up to R$ 2,994 Three minimum monthly salaries ☐ From R$ 2,995 to R$ 6,986 From three to seven salaries ☐ From R$ 6 987 to R$ 14 970 From seven to fifteen salaries ☐ Over R$ 14,971 More than fifteen salaries ☐ I prefer not to answer

8 Level of schooling ☐ No school/Some primary school ☐ Primary school graduate ☐ Some high school ☐ High school graduate/Some college ☐ College degree

**Newborn Screening Questionnaire**

1 How important do you feel it is to do newborn screening? ☐ Very important 5 ☐ 4 ☐ 3 ☐ 2 ☐ 1/0 No important

2 Which option best describes newborn screening? ☐ Collection of a few drops of blood from the baby’s foot for early diagnosis of genetic and/or metabolic illnesses. ☐ Footprints taken at the birth center/hospital right after birth ☐ Collection of genetic material from the baby’s foot to detect Down Syndrome

3 Did the hospital explain the purpose of this screening? ☐ It was clearly explained ☐ It was more or less clearly explained ☐ It was not explained very clearly ☐ The purpose was not explained

4 Did your child do newborn screening? ☐ Yes, the hospital provided orientation and asked me about it before doing it ☐ Yes, the hospital did it automatically without asking me ☐ No ☐ I don’t know/I don’t recall

5 Did you seek out information on newborn screening beyond what was explained by the hospital? Where? ☐ Healthcare professional ☐ Hospital ☐ Press in general ☐ Specialized websites ☐ Social media ☐ Other parents ☐ I did not seek out information

6 When was newborn screening done? ☐ On the day of birth ☐ One day after birth ☐ Two to five days after birth ☐ One week after birth ☐ One month or more after birth ☐ Do not remember ☐ Other

7 Had your child already breastfed for the first time or taken formula when newborn screening was administered? ☐ Yes ☐ No ☐ I don’t know/I don’t recall

8 Was the expanded newborn screening offered? ☐ Yes, but I refused it ☐ Yes and I had it done ☐ It was not offered ☐ I don’t know what expanded newborn screening is

9 How long after collection did you receive your newborn screening results? ☐ Up to 15 days after collection ☐ 16 to 30 days after collection ☐ 31 to 60 days after collection ☐ Over two months after collection ☐ I did not receive the results ☐ I don’t remember

10 Have you ever heard about the main diseases identified using newborn screening? Please indicate whether you have heard about any of the diseases below. ☐ Biotinidase Deficiency ☐ Sickle Cell Disease ☐ Phenylketonuria (PKU) ☐ Cystic fibrosis ☐ Congenital adrenal hyperplasia ☐ Congenital hypothyroidism ☐ None of these ☐ I don’t know

11 Did you know that newborn screening is available in Brazilian public health system and is offered free of charge to everyone?☐ Yes ☐ No

12 Did you refuse newborn screening? ☐ Yes, because I feel the baby suffers ☐ Yes, because I was not advised and I was afraid to do it ☐ Yes, because I see no advantages to doing the exam ☐ No, I did not refuse it

13 Did your child’s pediatrician see newborn screening at any time? ☐ Yes ☐ No ☐ I don’t remember

14 Did your child’s newborn screening show any abnormalities? ☐ Yes ☐ No

15 Who contacted you to notify that newborn screening had shown a positive/abnormal result? ☐ A recognized newborn screening service ☐ Hospital where my child was born ☐ Pediatrician ☐ Social worker ☐ Someone else

16 To what degree would you say that the person who contacted you clearly explained the diagnosis and what the next steps were? ☐ It was clearly explained ☐ It was more or less clearly explained ☐ It was not explained very clearly ☐ Nothing was explained and we were referred to the recognized newborn screening service in my city.

**PKU Questionnaire**

- **Original Portuguese version**

**Informações gerais**

1. Qual é a sua relação com a fenilcetonúria? ☐ Tenho fenilcetonúria ☐ Sou pai, mãe ou responsável por alguém com fenilcetonúria

2. O paciente é ☐ mulher ☐ homem

3. Em qual região do país você mora? ☐ Norte ☐ Nordeste ☐ Centro-Oeste ☐ Sudeste ☐ Sul

4. Renda familiar ☐ Até R$ 2 994 Três salários mínimos ☐ De R$ 2 995 a R$ 6 986 De três a sete salários mínimos ☐ De R$ 6 987 a R$ 14 970 De sete a quinze salários mínimos ☐ Acima de R$ 14 971 Mais de quinze salários mínimos ☐ Prefiro não responder

5. Escolaridade ☐ Nunca foi à escola ☐ Analfabeto/Fundamental incompleto (parou entre o 1º e o 4º ano) ☐ Fundamental I completo (fez até o 5º ano) Fundamental II incompleto (parou entre o 6º e o 8º ano) ☐ Fundamental completo (fez até o 9º ano) Médio incompleto (parou entre o 1º e o 2º colegial) ☐ Médio completo (fez até o 3º colegial) Superior incompleto (começou a faculdade/graduação) ☐ Superior completo (terminou a faculdade/graduação)

**Questionário**

1. Com qual idade ocorreu o diagnóstico da fenilcetonúria? ☐ Recém-nascido ☐ Entre 1 e 5 anos de idade ☐ Acima de 10 anos de idade

2. Você sabe o motivo de ter ocorrido o diagnóstico tardio? Base: 23 (apenas para quem teve diagnóstico tardio) ☐ O teste do pezinho apresentou resultado normal/negativo para fenilcetonúria ☐ Não foi feito o teste do pezinho na maternidade ☐ O resultado do teste do pezinho não foi recebido ☐ Falta de conhecimento dos médicos ☐ Outros motivos

3. Qual é a sua idade atual ou a da pessoa que tem fenilcetonúria? ☐ Até 2 anos de idade ☐ De 3 a 5 anos de idade ☐ De 6 a 10 anos de idade ☐ De 11 a 15 anos de idade ☐ De 16 a 20 anos de idade ☐ De 21 a 30 anos de idade ☐ Acima de 31 anos de idade

4. O pediatra solicitou o resultado do teste do pezinho em alguma consulta de rotina? Base: 21 (apenas cuidadores de pessoas com diagnóstico tardio) ☐ Não ☐ Sim

5. Qual palavra melhor descreve o momento do diagnóstico? ☐ Aceitação ☐ Angústia ☐ Ansiedade ☐ Choque ☐ Culpa ☐ Desafio ☐ Fé ☐ Negação ☐ Medo ☐ Revolta ☐ Tristeza ☐ Outro

6. Numa escala de 0 a 5, no momento do diagnóstico o médico esclareceu o que era a doença, os cuidados necessários e tirou suas dúvidas? ☐ 0 Não explicou/não esclareceu nada ☐ 1 ☐ 2 ☐ 3 ☐ 4 ☐ 5 Explicou/esclareceu completamente

7. Como foi a reação ao diagnóstico? ☐ Solicitei um novo exame para confirmação ☐ Não acreditei e fui procurar outro médico ☐ Fui pesquisar na internet para entender melhor o que é e como me adaptar a fenilcetonúria ☐ Fui procurar ajuda psicológica ☐ Fui procurar ajuda espiritual/religiosa ☐ Demorei para conseguir contar para meus familiares ☐ Entrei em depressão ☐ Tive crise de ansiedade

8. De quanto em quanto tempo é feito o exame de sangue para checar os níveis de fenilalanina no sangue? ☐ Toda semana ☐ A cada quinze dias ☐ Uma vez por mês ☐ A cada três meses ☐ Uma vez por ano ☐ Nunca/raramente

9. Que quadros abaixo, e em que intensidade, você ou o paciente apresenta? (em ordem de impacto no paciente)

Déficit de atenção ou dificuldade na escola ☐ Extrema ☐ Alta ☐ Média ☐ Baixa ☐ Sem sintomas

Atraso intelectual ☐ Extrema ☐ Alta ☐ Média ☐ Baixa ☐ Sem sintomas

Problemas comportamentais ou sociais ☐ Extrema ☐ Alta ☐ Média ☐ Baixa ☐ Sem sintomas

Comprometimento neurológico grave ☐ Extrema ☐ Alta ☐ Média ☐ Baixa ☐ Sem sintomas

Dermatite ou outras manifestações na pele ☐ Extrema ☐ Alta ☐ Média ☐ Baixa ☐ Sem sintomas

Obesidade ☐ Extrema ☐ Alta ☐ Média ☐ Baixa ☐ Sem sintomas

Convulsões, tremores ou movimentos espasmódicos nos braços e pernas ☐ Extrema ☐ Alta ☐ Média ☐ Baixa ☐ Sem sintomas

Alterações no crescimento ☐ Extrema ☐ Alta ☐ Média ☐ Baixa ☐ Sem sintomas

Desnutrição ☐ Extrema ☐ Alta ☐ Média ☐ Baixa ☐ Sem sintomas

10. Com que intensidade se manifestam os sintomas abaixo?

Ansiedade ☐ Extrema ☐ Alta ☐ Média ☐ Baixa ☐ Sem sintomas

Irritabilidade ☐ Extrema ☐ Alta ☐ Média ☐ Baixa ☐ Sem sintomas

Dificuldades de organização/ planejamento ☐ Extrema ☐ Alta ☐ Média ☐ Baixa ☐ Sem sintomas

Falta de concentração ☐ Extrema ☐ Alta ☐ Média ☐ Baixa ☐ Sem sintomas

Impulsividade ☐ Extrema ☐ Alta ☐ Média ☐ Baixa ☐ Sem sintomas

Dificuldades para raciocinar ☐ Extrema ☐ Alta ☐ Média ☐ Baixa ☐ Sem sintomas

Dificuldade de compreensão ☐ Extrema ☐ Alta ☐ Média ☐ Baixa ☐ Sem sintomas

Problemas de memória ☐ Extrema ☐ Alta ☐ Média ☐ Baixa ☐ Sem sintomas

Distúrbios do sono (insônia, apneia) ☐ Extrema ☐ Alta ☐ Média ☐ Baixa ☐ Sem sintomas

Agressividade ☐ Extrema ☐ Alta ☐ Média ☐ Baixa ☐ Sem sintomas

Fobias ☐ Extrema ☐ Alta ☐ Média ☐ Baixa ☐ Sem sintomas

11. Como é feito o controle dos níveis de fenilalanina na rotina? ☐ Peso diariamente os alimentos de todas as refeições, faço o cálculo da quantidade de fenil de acordo com o permitido por dia passado pela nutricionista juntamente com a quantidade de fórmula ☐ Peso sempre que possível os alimentos, mas não consigo realizar o cálculo da quantidade de fenil para todas as refeições nem sempre administro a fórmula ☐ Não consigo pesar os alimentos nem fazer o cálculo da quantidade de fenil, mas tenho experiência em gerenciar a dieta e administro a fórmula quando sinto que meu familiar precisa tomar ☐ Não faço muito controle, pois vejo que meu filho/familiar se sente bem e posso flexibilizar a dieta. ☐

12. Quanto você acha que a fenilcetonúria está controlada? ☐ Totalmente controlada 5 ☐ 4 ☐ 3 ☐ 2 ☐ 1 Nada controlada

13. Qual é a maior dificuldade para seguir com o tratamento? ☐ Não há alimentos hipoproteicos disponíveis na rede pública ☐ Mudança frequente nas marcas de fórmula de aminoácidos fornecida pela rede pública ☐ Seguir e controlar diariamente a dieta devido ao nível de restrição ☐ Não há medicamento para tratamento da fenilcetonúria ☐ Não consigo encontrar com facilidade os alimentos sugeridos pelo nutricionista ☐ Ter uma alimentação diferente do restante da família ☐ O medicamento para o tratamento da fenilcetonúria não é disponibilizado pela rede pública ☐ Exclusão social/ falta de compreensão das pessoas ☐ Fazer o exame de sangue em todas as consultas ☐ Dificuldade de deslocamento até a consulta/falta de transporte adequado ☐ Pouca compreensão e apoio de familiares e amigos ☐ Dificuldade para compreender as orientações do médico ou nutricionista

14. Já foi feito algum teste neuropsicológico para avaliar os impactos da fenilcetonúria?

QI ☐ Sim, mais de uma vez ☐ Sim, apenas uma vez ☐ Não, nunca foi feita nenhuma avaliação desse tipo ☐ O médico comentou a respeito, mas o teste não está disponível na rede pública ou no SRTN e não tenho como arcar com essas despesas

Cognição ☐ Sim, mais de uma vez ☐ Sim, apenas uma vez ☐ Não, nunca foi feita nenhuma avaliação desse tipo ☐ O médico comentou a respeito, mas o teste não está disponível na rede pública ou no SRTN e não tenho como arcar com essas despesas

Função executiva ☐ Sim, mais de uma vez ☐ Sim, apenas uma vez ☐ Não, nunca foi feita nenhuma avaliação desse tipo ☐ O médico comentou a respeito, mas o teste não está disponível na rede pública ou no SRTN e não tenho como arcar com essas despesas

15. Quanto você consegue seguir à risca as orientações do médico? ☐ Sigo todas as orientações ☐ Sigo a maioria das orientações aproximadamente ☐ Meu médico não faz muitas orientações, recebo a maior parte das orientações pelo nutricionista ☐ Sigo a metade das orientações aproximadamente ☐ Sigo poucas orientações

16. Quanto você consegue seguir à risca as orientações do nutricionista? ☐ Sigo todas as orientações ☐ Sigo a maioria das orientações aproximadamente ☐ Sigo a metade das orientações aproximadamente ☐ Sigo poucas orientações ☐ Não sigo nada ☐ Não tenho acompanhamento regular do nutricionista

17. A fenilcetonúria teve impacto financeiro na sua vida? ☐ Sim, pois tenho alto gasto com consultas e terapias e compra de alimentos especiais ☐ Sim, precisei parar de exercer minhas atividades profissionais para cuidar do meu filho ☐ Sim, pois pessoas da minha família deixaram de trabalhar para me auxiliar ou para auxiliar o familiar com a doença ☐ Sim, pois tive que contratar uma pessoa para me auxiliar ☐ Não, pois tenho uma situação financeira confortável e a doença não impactou minha renda

18. Já houve algum tipo de preconceito devido à fenilcetonúria? ☐ Sim ☐ Não

19. Como foi sua adaptação ou a da pessoa de quem você cuida na escola?

Professores e funcionários entenderam e ajudaram na manutenção da dieta e no tratamento ☐ Sim ☐ Não

Houve discriminação por parte dos pais dos colegas por causa das restrições alimentares ☐ Sim ☐ Não

Houve discriminação por parte dos colegas, não queriam conviver, não convidavam para festas, não entenderam as restrições alimentares ☐ Sim ☐ Não

Pouca colaboração dos professores e pais de outros colegas. ☐ Sim ☐ Não

Perdi boa parte do controle sobre a dieta e percebi alterações no comportamento ☐ Sim ☐ Não

20. Como a fenilcetonúria afeta ou atrapalha

A autoestima ☐ Não afeta nada ☐ Afeta com baixa intensidade ☐ Afeta com média intensidade ☐ Afeta com alta intensidade ☐ Afeta com extrema intensidade

A convivência familiar ☐ Não afeta nada ☐ Afeta com baixa intensidade ☐ Afeta com média intensidade ☐ Afeta com alta intensidade ☐ Afeta com extrema intensidade

Atividades sociais com amigos, ir a festas, restaurantes e participação em datas comemorativas ☐ Não afeta nada ☐ Afeta com baixa intensidade ☐ Afeta com média intensidade ☐ Afeta com alta intensidade ☐ Afeta com extrema intensidade

Dedicação da pessoa de quem você cuida na escola ☐ Não afeta nada ☐ Afeta com baixa intensidade ☐ Afeta com média intensidade ☐ Afeta com alta intensidade ☐ Afeta com extrema intensidade

A sua vida profissional ☐ Não afeta nada ☐ Afeta com baixa intensidade ☐ Afeta com média intensidade ☐ Afeta com alta intensidade ☐ Afeta com extrema intensidade

21. Indique quanto cada profissional/meio de comunicação o apoia no dia a dia e no tratamento da fenilcetonúria (em ordem de impacto)

Nutricionista ☐ Apoia muito ☐ Apoia um pouco ☐ Não apoia ☐ Não se aplica

Família ☐ Apoia muito ☐ Apoia um pouco ☐ Não apoia ☐ Não se aplica

Médico ☐ Apoia muito ☐ Apoia um pouco ☐ Não apoia ☐ Não se aplica

Grupos de pacientes ☐ Apoia muito ☐ Apoia um pouco ☐ Não apoia ☐ Não se aplica

Redes sociais ☐ Apoia muito ☐ Apoia um pouco ☐ Não apoia ☐ Não se aplica

Psicólogo ☐ Apoia muito ☐ Apoia um pouco ☐ Não apoia ☐ Não se aplica

Rede pública ☐ Apoia muito ☐ Apoia um pouco ☐ Não apoia ☐ Não se aplica

Governo ☐ Apoia muito ☐ Apoia um pouco ☐ Não apoia ☐ Não se aplica

22. Quanto você acredita que o Brasil está preparado para atender o paciente com fenilcetonúria? ☐ Muito preparado ☐ Um pouco preparado ☐ Nada preparado

- **English version**

**General information**

1 What is your relationship with phenylketonuria? ☐ I have phenylketonuria ☐ I am the father, mother or guardian of someone withphenylketonuria

2 The patient is: ☐ women ☐ men

3 Which Brazilian region do you live in? ☐ North ☐ Northeast ☐ Midwest ☐ Southeast ☐ South

4 Family income ☐ Up to R$ 2 994 Three minimum monthly salaries ☐ From R$ 2 995 to R$ 6 986 From three to seven salaries ☐ From R$ 6 987 to R$ 14 970 From seven to fifteen salaries ☐ Above R$ 14 971 More than fifteen salaries ☐ I prefer not to answer

5 Patient’s level schooling ☐ No school ☐ Illiterate/Some primary school ☐ Primary school graduate ☐ Some high school ☐ High school graduate/Some college ☐ College degree

**Questionnaire**

1 At what age was phenylketonuria diagnosed? ☐ Newborn ☐ Between 1-5 years old ☐ Over 10 years old

2 Do you know why it was diagnosed late? ☐ The results of newborn screening were normal/ negative for phenylketonuria ☐ Newborn screening was not done at the birth center ☐ Newborn screening results were not received ☐ Lack of medical knowledge ☐ A different reason

3 What is your current age or phenylketonuria patient age? ☐ Under 2 years old ☐ 3-5 years old ☐ 6 to 10 years old ☐ 11 to 15 years old ☐ 16 to 20 years old ☐ 21 to 30 years old ☐ Over 31 years old

4 Did the pediatrician ask for the results of newborn screening during any routine appointment? ☐ Yes ☐ No ☐

5 What word best describes the moment of diagnosis? ☐ Acceptance ☐ Anguish ☐ Anxiety ☐ Challenge ☐ Denial ☐ Depression ☐ Faith ☐ Fear ☐ Guilt ☐ Revolt ☐ Sadness ☐ Shock ☐ Other

6 On a scale of 0 to 5, upon diagnosis, did the doctor explain what the disease was, discuss the care needed and answer your questions? ☐ 5 It was fully explained/clarified ☐ 4 ☐ 3 ☐ 2 ☐ 1 ☐ 0 Nothing was explained/clarified

7 What was your reaction to the diagnosis? ☐ I asked for a new exam to confirm ☐ I didn’t believe it and went to another doctor ☐ I researched online to gain a better understanding of what it is and how to adapt to phenylketonuria ☐ I sought out psychological help ☐ I sought out spiritual/religious help ☐ I took some time to be able to tell my family ☐ I became depressed ☐ I had a panic attack

8 How often is a blood test done to check phenylalanine levels? ☐ Every week ☐ Every fifteen days ☐ Once a month ☐ Every three months ☐ Once a year ☐ Never/rarely

9 What conditions below do you or the patient have? (in order of impact on the patient)

Attention deficit or academic difficulties ☐ Extremely intense ☐ Very intense ☐ More or less intense ☐ Somewhat intense ☐ Not present

Intellectual impairment ☐ Extremely intense ☐ Very intense ☐ More or less intense ☐ Somewhat intense ☐ Not present

Behavioral or social problems ☐ Extremely intense ☐ Very intense ☐ More or less intense ☐ Somewhat intense ☐ Not present

Severe neurological impairment ☐ Extremely intense ☐ Very intense ☐ More or less intense ☐ Somewhat intense ☐ Not present

Dermatitis or other skin manifestations ☐ Extremely intense ☐ Very intense ☐ More or less intense ☐ Somewhat intense ☐ Not present

Obesity ☐ Extremely intense ☐ Very intense ☐ More or less intense ☐ Somewhat intense ☐ Not present

Convulsions, tremors or spasmodic movements in the arms and legs ☐ Extremely intense ☐ Very intense ☐ More or less intense ☐ Somewhat intense ☐ Not present

Changes in growth ☐ Extremely intense ☐ Very intense ☐ More or less intense ☐ Somewhat intense ☐ Not present

Malnutrition ☐ Extremely intense ☐ Very intense ☐ More or less intense ☐ Somewhat intense ☐ Not present

10 How intensely do the symptoms below manifest? (in order of impact on the patient)

Anxiety ☐ Extremely intense ☐ Very intense ☐ More or less intense ☐ Somewhat intense ☐ Not present

Irritability ☐ Extremely intense ☐ Very intense ☐ More or less intense ☐ Somewhat intense ☐ Not present

Difficulties with organizing/planning ☐ Extremely intense ☐ Very intense ☐ More or less intense ☐ Somewhat intense ☐ Not present

Lack of concentration ☐ Extremely intense ☐ Very intense ☐ More or less intense ☐ Somewhat intense ☐ Not present

Impulsiveness ☐ Extremely intense ☐ Very intense ☐ More or less intense ☐ Somewhat intense ☐ Not present

Difficulty thinking ☐ Extremely intense ☐ Very intense ☐ More or less intense ☐ Somewhat intense ☐ Not present

Difficulty understanding ☐ Extremely intense ☐ Very intense ☐ More or less intense ☐ Somewhat intense ☐ Not present

Memory problems ☐ Extremely intense ☐ Very intense ☐ More or less intense ☐ Somewhat intense ☐ Not present

Sleep disorders (insomnia, apnea) ☐ Extremely intense ☐ Very intense ☐ More or less intense ☐ Somewhat intense ☐ Not present

Aggressiveness ☐ Extremely intense ☐ Very intense ☐ More or less intense ☐ Somewhat intense ☐ Not present

Phobias ☐ Extremely intense ☐ Very intense ☐ More or less intense ☐ Somewhat intense ☐ Not present

11 How are phenylalanine levels routinely controlled? ☐ Food for all meals is weighed daily, I calculate the amount of phenyl according to the daily allowance provided by the nutritionist along with the amount of formula ☐ Food is weighed whenever possible, but I’m unable to calculate the amount of phenyl for all meals and do not always administer the formula ☐ I am neither able to weigh food nor do the calculation of the amount of phenyl, but I am experienced at diet management and I administer the formula when I feel that my family member needs to take it ☐ ☐ Not much control is done, since I see that my child/family member feels well and I can make the diet flexible

12 How controlled do you feel the phenylketonuria is? ☐ 5 Totally controlled ☐ 4 ☐ 3 ☐ 2 ☐ 1 Not controlled at all

13 What is the greatest obstacle to continued treatment? ☐ The public health system does not provide low-protein foods ☐ Frequently changing amino acid formulas provided by the public health system ☐ Following and controlling diet on a daily basis, due to the level of restrictions ☐ There is no medication treating phenylketonuria ☐ I am unable to easily find the food suggested by the nutritionist ☐ Having a different diet from the rest of the family ☐ The medication available to treat phenylketonuria is not available from the public health system ☐ Social exclusion/ lack of understanding by others ☐ Daily weighing of all food ☐ Doing a blood test at every doctor’s visit ☐ Difficulty getting to the doctor’s office/ lack of appropriate transportation ☐ Little understanding and support from family and friends ☐ Difficulty understanding the orientation provided by the physician or nutritionist

14 Has neuropsychological testing ever been done to assess the impacts of phenylketonuria? ☐ Yes, more than once ☐ Yes, only once ☐ No, I’ve never done any test of this kind ☐ The doctor mentioned it, but the test is not available in the public health system or from the Neonatal Screening Center and I have no way to cover these costs

15 How closely are you or the patient you care for able to follow the doctor’s orientations? ☐ I follow all of them ☐ I follow most of them approximately ☐ I follow half of them approximately ☐ My doctor does not provide many orientations; I receive most of them from my nutritionist ☐ I follow very few of them

16 How closely are you able to follow the nutritionist’s orientations? ☐ I follow all of them ☐ I follow most of them approximately ☐ I follow half of them approximately ☐ I follow very few of them ☐ I don’t follow them at all ☐ I don’t regularly see a nutritionist

17 Has phenylketonuria impacted your life financially? ☐ Yes, because I have incurred significant costs from doctor visits, therapy and the purchase of special food ☐ Yes, I had to stop working to take care of my child ☐ Yes, because people in my family have stopped working to help me or to help our family member with the disease ☐ Yes, because I had to hire someone to help me ☐ No, because I am financially comfortable and the disease has not impacted my income

18 Have you ever suffered any kind of prejudice because of phenylketonuria? ☐ Yes ☐ No

19 What was it like for you or the person you care for to adapt to school?

Teachers and employees were understanding and helped to maintain diet and treatment ☐ Yes ☐ No

There was discrimination by classmates’ parents because of food restrictions ☐ Yes ☐ No

Classmates discriminated and were not friendly, did not invite me to parties, did not understand diet restrictions ☐ Yes ☐ No

Little collaboration from teachers and classmates’ parents. I lost a lot of control over diet and noticed behavioral changes ☐ Yes ☐ No

20 How phenylketonuria affects/hinders:

Self-esteem ☐ No affect at all ☐ Low intensity affect ☐ Medium intensity affect ☐ High intensity affect ☐ Extremely intense affect

Family life ☐ No affect at all ☐ Low intensity affect ☐ Medium intensity affect ☐ High intensity affect ☐ Extremely intense affect

Social activities with friends, going to parties, restaurants and participating in commemorative dates ☐ No affect at all ☐ Low intensity affect ☐ Medium intensity affect ☐ High intensity affect ☐ Extremely intense affect

Dedication at school ☐ No affect at all ☐ Low intensity affect ☐ Medium intensity affect ☐ High intensity affect ☐ Extremely intense affect

Professional life ☐ No affect at all ☐ Low intensity affect ☐ Medium intensity affect ☐ High intensity affect ☐ Extremely intense affect

21 Please indicate how much each professional/means of communication supports you on a daily basis and in treating phenylketonuria.

Nutritionist ☐ Highly supportive ☐ Somewhat supportive ☐ Not supportive ☐ Don’t use/Does not apply

Family ☐ Highly supportive ☐ Somewhat supportive ☐ Not supportive ☐ Don’t use/Does not apply

Physician ☐ Highly supportive ☐ Somewhat supportive ☐ Not supportive ☐ Don’t use/Does not apply

Patient Groups ☐ Highly supportive ☐ Somewhat supportive ☐ Not supportive ☐ Don’t use/Does not apply

Social Media ☐ Highly supportive ☐ Somewhat supportive ☐ Not supportive ☐ Don’t use/Does not apply

Psychologist ☐ Highly supportive ☐ Somewhat supportive ☐ Not supportive ☐ Don’t use/Does not apply

Public Health System ☐ Highly supportive ☐ Somewhat supportive ☐ Not supportive ☐ Don’t use/Does not apply

Government ☐ Highly supportive ☐ Somewhat supportive ☐ Not supportive ☐ Don’t use/Does not apply

22 How prepared do you feel Brazil is to serve patients with phenylketonuria? ☐ Very prepared ☐ Somewhat prepared ☐ Not at all prepared

**Results**

**Table 3.** General answers of all parents in NBS questionnaire.

|  | **All participants (%)**  **n = 1000** |
| --- | --- |
| **Importance of NBS** |  |
| Very important 5 | 93 |
| 4 | 3 |
| 3 | 2 |
| 2 | 2 |
| 1/0 No important | 1 |
| **Best description of the NBS** |  |
| Collection of a few drops of blood from the baby's foot for early diagnosis of genetic and/or metabolic illnesses. | 80 |
| Footprints taken at the birth center/hospital right after birth | 12 |
| Collection of genetic material from the baby's foot to detect Down Syndrome | 8 |
| **Hospital explanation about NBS purpose** |  |
| It was clearly explained | 46 |
| It was more or less clearly explained | 25 |
| It was not explained very clearly | 11 |
| The purpose was not explained | 18 |
| **Search for more information about the NBS beyond what the hospital offered** |  |
| Healthcare professional | 25 |
| Hospital | 13 |
| Press in general | 5 |
| Specialized websites | 27 |
| Social media | 15 |
| Other parents | 18 |
| I did not seek out information | 35 |
| **Knowledge about the main diseases detected by the NBS** |  |
| Biotinidase Deficiency | 13 |
| Sickle cell disease | 21 |
| Phenylketonuria (PKU) | 12 |
| Cystic fibrosis | 19 |
| Congenital adrenal hyperplasia | 14 |
| Congenital hypothyroidism | 19 |
| None of these | 10 |
| I don't know | 53 |
| **Knowledge that NBS is free of charge on the public healthcare system to all live births in Brazil** |  |
| Yes | 84 |
| No | 16 |

**Table 4.** Emotional and symptomatic results of the PKU questionnaire.

|  | **Patients, parents and caregivers (%)** | | | | |
| --- | --- | --- | --- | --- | --- |
| **PKU Affects or hinders. n = 228** | **Extremely intense affect** | **High intensity affect** | **Medium intensity affect** | **Low intensity affect** | **No affect at all** |
| Self-esteem | 4 | 5 | 14 | 30 | 47 |
| Family life | 0 | 4 | 12 | 23 | 61 |
| Social activities with friends, going to parties, restaurants and participating in commemorative dates | 10 | 15 | 21 | 29 | 25 |
| Dedication at school | 4 | 4 | 11 | 20 | 61 |
| Professional life | 13 | 10 | 13 | 21 | 43 |
| **Impact of PKU. n = 228** | **Extremely intense** | **Very intense** | **More or less intense** | **Somewhat intense** | **Not present** |
| Attention deficit or academic difficulties | 6 | 8 | 14 | 15 | 57 |
| Intellectual impairment | 3 | 4 | 7 | 7 | 79 |
| Behavioral or social problems | 2 | 4 | 7 | 7 | 74 |
| Severe neurological impairment | 2 | 2 | 4 | 4 | 88 |
| Dermatitis or other skin manifestations | 1 | 8 | 8 | 18 | 65 |
| Obesity | 1 | 1 | 7 | 7 | 84 |
| Convulsions, tremors or spasmodic movements in the arms and legs | 0 | 1 | 4 | 8 | 87 |
| Changes in growth | 0 | 0 | 5 | 9 | 86 |
| Malnutrition | 0 | 1 | 1 | 7 | 91 |
| **Intensity of PKU symptoms. n =228** | **Extremely intense** | **Very intense** | **More or less intense** | **Somewhat intense** | **Not present** |
| Anxiety | 7 | 16 | 20 | 24 | 33 |
| Irritability | 7 | 12 | 23 | 36 | 22 |
| Difficulties with organizing/planning | 9 | 8 | 13 | 20 | 50 |
| Lack of concentration | 4 | 12 | 20 | 22 | 42 |
| Impulsiveness | 5 | 8 | 17 | 19 | 51 |
| Difficulty thinking | 6 | 7 | 14 | 16 | 57 |
| Difficulty understanding | 4 | 8 | 15 | 20 | 53 |
| Memory problems | 3 | 6 | 9 | 16 | 66 |
| Sleep disorders (insomnia, apnea) | 3 | 5 | 11 | 16 | 65 |
| Aggressiveness | 2 | 3 | 11 | 21 | 63 |
| Phobias | 0 | 5 | 6 | 17 | 72 |
| **Support by professionals/media on a daily basis and in the treatment of PKU (in order of impact) n = 228** | **Supports a lot** | **Supports a little** | **Does not support** | **Does not apply** | **-** |
| Nutritionist | 75 | 21 | 3 | 1 | - |
| Family | 71 | 27 | 1 | 1 | - |
| Physician | 58 | 29 | 6 | 7 | - |
| Patient groups | 56 | 24 | 3 | 17 | - |
| Social media | 47 | 42 | 4 | 7 | - |
| Psychologist | 38 | 23 | 7 | 32 | - |
| Public Health System | 25 | 48 | 19 | 8 | - |
| Government | 3 | 53 | 40 | 4 | - |
| **Neuropsychological test to assess the impacts of PKU n = 228** | **IQ** | **Cognition** | **Executive function** | - | - |
| Yes, more than once | 11 | 16 | 11 | - | - |
| Yes, only once | 14 | 10 | 12 | - | - |
| No, I've never done any test of this kind | 68 | 69 | 71 | - | - |
| The doctor mentioned it, but the test is not available in the public health system or from the Neonatal Screening Center and I have no way to cover these costs | 7 | 5 | 6 | - | - |
| **Adaptation at school n = 181 (only those who went to school)** | **Yes** | **No** | - | - | - |
| Teachers and employees were understanding and helped to maintain diet and treatment | 83 | 17 | - | - | - |
| There was discrimination by classmates' parents because of food restrictions | 19 | 81 | - | - | - |
| Classmates discriminated and were not friendly, did not invite me to parties, did not understand diet restrictions | 12 | 88 | - | - | - |
| Little collaboration from teachers and classmates' parents. I lost a lot of control over diet and noticed behavioral changes | 13 | 87 | - | - | - |

**Table 5**. Only parents and caregivers in PKU questionnaire

|  | **Only parents and caregivers (%)**  n = 195 |
| --- | --- |
| **Word that best describes the moment of diagnosis** |  |
| Shock | 32 |
| Fear | 28 |
| Sadness | 10 |
| Acceptance | 6 |
| Challenge | 5 |
| Faith | 3 |
| Revolt | 3 |
| Other | 3 |
| Depression | 3 |
| Anxiety | 2 |
| Denial | 2 |
| Guilt | 2 |
| Anguish | 1 |
| **Clarifications on the disease necessity of care at diagnosis** |  |
| 0 Nothing was explained/clarified | 7 |
| 1 | 2 |
| 2 | 7 |
| 3 | 12 |
| 4 | 10 |
| 5 It was fully explained/clarified | 62 |
| **Patient´s reaction to the diagnosis** |  |
| I asked for a new exam to confirm | 35 |
| I didn't believe it and went to another doctor | 7 |
| I researched online to gain a better understanding of what it is and how to adapt to phenylketonuria | 64 |
| I sought out psychological help | 7 |
| I sought out spiritual/religious help | 14 |
| I took some time to be able to tell my family | 6 |
| I became depressed | 14 |
| I had a panic attack | 24 |
| **Frequency of blood tests to check Phe blood levels** |  |
| Every week | 2 |
| Every fifteen days | 7 |
| Once a month | 44 |
| Every three months | 44 |
| Once a year | 3 |
| Never/rarely | 0 |
